# Cheap Artificial Teeth, and Cheap Dental Operations in England

**Published:** 1851-07

**Authors:** 


### ARTICLE IV.

### *Cheap Artificial Teeth, and Cheap Dental Operations in England.*

As observed in my last paper, the dental profession in England has, from the peculiar advantages it seems to hold out to indolence and rapacity, become a kind of *dernier resort*, for all those who from want of inclination for, or success in, the callings to which they were originally educated, have determined to abandon them for some other; and hence it is, that it is made up of a heterogeneous mass of individuals, drafted from all kinds of trades and professions, who have embraced it, not because they have any particular vocation for the art, or fitness for its practice, but simply that they consider it a light, easy and profitable profession, in which many have acquired fortunes without any remarkable amount of talent, and which in a common way makes few calls on the convenience or comfort of the practitioner.

It is scarcely surprising then that among the host of dentists who daily inundate the metropolis, many should be found, who, if ignorance be bliss, must be in a state of perfect beatitude; in short, whose whole qualifications for the profession consist in getting out in some way or other, a natural tooth, and making and adapting, after a somewhat rude fashion, *very* artificial ones, who know nothing of the scientific or surgical branches of the art, a perfect acquaintance with which forms the dentist, properly so called, and to use an Irishism, less of its physiology. These men generally commence practice without having received what, by any extension of courtesy, can be considered a professional education; their whole stock of trade consisting of a module of empyrical knowledge, with a more than tolerable share of impudence, and armed with this and aided by a series of five shilling advertisements, they sometimes crawl into a kind of disreputable practice. Even this, however, they could not effect, did they not bring cheapness to their aid, and offer to do for shillings that which a respectable practitioner could not, and would not, do under as many pounds, and this with the fact staring them in the face (for they cannot be so completely ignorant of the prices of the best materials though they never employ them) that putting artistic skill and manipulations out of the question, the expense of proper materials alone, would actually *cost them* more than they charge for every thing inclusive, and that a single tooth, or any number of teeth, cannot be made and properly finished but at a much higher price than they charge, even were they to give their valuable services and could live on the honor of operating, that there would actually be a greater outlay for material than the cheap, very cheap, dentists receive for their time, labor, skill, &c. Thus they must either cheat the public with promises, or must have the power, like the Irish huckster, to buy and sell and live by the loss.

But these gentlemen again, not content with supplying artificial grinders, at a price which, to use their own words, brings them within the most limited means, offer their services to stop teeth and perform various other operations on equally liberal terms. And some of these individuals not only offer to stop single teeth at 1s. 6d. each, but actually offer to do business wholesale, to take the whole mouth, and stop all the teeth that may require it, from three to two and thirty, at the very moderate charge of 10s.—while some more liberal still offer their wares and services at the same time, and sell the material for stopping, with such directions as will enable any man to plaster his own grinders, at 2s. 9d. stamp included. For the sake of contrast, I will place in juxta position, with one of these cheap men, a respectable practitioner who does not advertise, and who will not plug a tooth under the regular fee of 10s. 6d. to two guineas, or in other words, who will not perform services that require great skill, involve some responsibility, demand the sacrifice of an hour to two hours and an half, (to say nothing of material, which in some cases forms a very considerable item,) without a fair and proper remuneration; and I will ask which is likely to be the honest man, he who *professes* to work at somewhat about plasterer's wages, or he who consults the usefulness and dignity of his profession, and insists on such a remuneration as is consistent with his station as a gentleman, and sufficient to insure a proper performance of the operation.

Again, your Jones', Thomas' and Read's, cum multis aliis, *profess* to insert a single tooth for five shillings, nay, for three and sixpence, and decorate the whole mouth with a complete set of grinders, warranted to divide a spoonful of calves-foot jelly, for the small sum of five pound. While the non-advertising portion of the profession will not insert a complete set for less than twenty-five or thirty pounds, or in other words, four or five times as much as these cheap dental costermongers.

All this of course is understood by the profession, who know what the material costs, and what is and what is not, a fair remuneration for skill and sufficient to ensure competency and honesty; but with the public the case is very different, and a non-professional individual, who is apt to class all dentists in the same category, must be somewhat puzzled to know how one person can venture to charge twenty-five pounds for services and material, which another offers to supply for five pounds, and that, according to his advertisement, in the best manner and of the best materials. He either concludes that in the one case he is paying for *reputation* rather than talent, or like an ass between two panniers cannot tell which side to turn, but fancying one of them must be a swindle, cannot decide whether it is that the cheap man cannot perform the services in the manner or at the price he promises, or that the dearer one charges most exorbitantly, for the commodity of the teeth on the one hand, and for his services in plugging them on the other—and as in the estimation of the ignorant, teeth, whether cut out of the hoof of a horse or the horn of the hippopotamus, whether manufactured of the same crockery as his cups and saucers or emanating from the laboratory of a Stockton or an Alcock, are still but teeth ; as in his estimation the metal, whether gilt copper, pinchback or standard gold, still comes under his denomination of gold, and as he can scarcely tell the difference, whether the work be of the highest dental finish or about equal to the manufacture of a cart wheel, the chances are that he gives a verdict in favor of imposition, and adds another to the gulls of cheap dentism.

That such would be the opinion of nine out of ten who read the cheap dental advertisements, and compare their prices with those of the more honorable and legitimate practitioner, I have no doubt, nor is it surprising that it should be so ; the public, generally speaking, are not apt to reason much on these matters, and in most cases are not aware that one practitioner is more respectable or honest than the other ; they are both members of the same profession, both dentists, perhaps both *surgeon* dentists.

Could any respectable member of the profession place himself in the same relative circumstances as regards any other trade, (for, as is generally practiced in England, it is honored by being called such,) I feel convinced that he would arrive at precisely the same conclusion. But let the non-professional really try the difference practically, and he will no longer feel surprise at the low charges of the one party, or the more ample ones of the other—unless he has a preference for the taste of copper-ore, especial delight in being salivated, or thinks it a luxury to have his mouth filled with coarse crockery, or animal matter that decomposes as rapidly as fish bone. R.

[To be continued.]

